# Notes and News

**Published:** 1899-02-25

**Authors:** 


### Notes and News.

(Collected and Communicated.)

HOSPITAL MEETINGS.

New Hospital for Women.—Annual Meeting, Tuesday, March 7th, at three o'clock p.m., at the Hospital.
Queen Charlotte's Lying-in Hospital.—Annual Meeting, Monday, February 27th, at half-past four o'clock p.m., at the Hospital.

At the Brompton Hospital for Consumption the outdoor treatment of the disease is about to be introduced.

A festival dinner in aid of the East London Hospital for Children, Shadwell, will be held on Monday, May 15th, at the Whitehall Rooms, Hotel Metropole, under the presidency of the Duke of Connaught.

Sir John Stirling Maxwell, Bart., M.P., has consented to preside at a festival dinner in aid of the funds of University College Hospital at the Whitehall Rooms, Hotel Metropole, on Thursday, June 15th next.

Small-pox was very prevalent in Johannesburg last year. The percentage of deaths was 25·7 amongst Europeans and 29·2 amongst the coloured patients. A special note is made of the fact that no deaths occurred amongst persons vaccinated within the last seven years.

America is constantly setting examples in sanitation. At one police-court in New York the taking of oaths has been rendered sanitary by the adoption of a celluloid cover to the Bible in use, which makes it possible to cleanse it by means of antiseptics.

Dr. Sims Woodhead, who has been elected Professor of Pathology at Cambridge, is well-known as an original worker. He has been many years Director of the Pathological Laboratories of the Royal Colleges of Physicians and Surgeons, at the Examination Hall on the Embankment.

Boards of Guardians are not as a rule remarkable for the display of energy to adopt new methods or reforms in the institutions under their control. It is, therefore, all the more surprising that the subject of tuberculosis has stirred them all over the country to consider the establishment of sanatoria, where the poor suffering from consumption can be isolated and the outdoor treatment applied.

Dr. James Taylor, M.A., M.D., F.R.C.P., Assistant Physician to the National Hospital for the Paralysed and Epileptic, and Physician to the North-Eastern Hospital for Children, has been appointed Physician to the Royal London Ophthalmic Hospital, Moorfields.

It appears that every facility for demonstrating the present condition of medicine and sanitary science is to be given at the Paris Exhibition of 1900. Exhibitors are invited to get together historical representations of treatment of the sick in different epochs, and to collect objects of interest and matters relating to the subject. As the railways are offering considerably reduced charges for the conveyance of exhibits, it is to be hoped this section will be fully represented.

A Medical, Surgical, and Hygienic Exhibition will take place at the Queen's Hall, Langham Place, W., this year, from May 23rd to 26th inclusive. The object of this exhibition (which is held under the auspices of the Medical, Surgical, and Hygienic Exhibitors' Association) is to show the progress made in all branches of medical science, as far as new instruments, drugs, and kindred preparations are concerned. Mr. Henry Blau, 29, New Bridge Street, London, E.C., is the hon. secretary.

The trustees of the Reid Trust for the Education of Women Fund have decided to offer a scholarship at the London (Royal Free Hospital) School of Medicine for Women in memory of their valued co-trustee, Miss Bostock, of Penmaen, Glamorganshire, lately deceased. The value of the scholarship will be £60 a year, tenable for two or four years, and awarded on the result of the preliminary scientific examination of the University of London. The Bostock scholar must read for the London medical degree. Further particulars may be obtained from the Hon. Secretary of the Reid Trust, Bedford College, York Place, W.

The third session of the International Congress of Gynæcology and Obstetrics will be held at Amsterdam from August 8th to 12th, 1899. The questions arranged for discussion are as follows: (1) The surgical treatment of fibro-myoma; (2) the relative value of antisepsis and improved technic for the actual results in gynæcological surgery; (3) the influence of posture on the form and dimensions of the pelvis; and (4) the indication for cæsarian section compared to that for symphyseotomy, craniotomy, and premature induction of labour. Among those who have consented to take part in these discussions are Messrs. Doyen, Howard Kelly, and Schauta, who will treat the first question; Messrs. Bumm, Richelot, and Lawson Tait the second; Messrs. Bonnaire, Pinzani, and Walcher the third; and Messrs. Leopold, Pinard, and Pestalozza the fourth. The reports, with their translations in the official languages, will be sent to all the members a month before the opening of the Congress. As regards private communications, preference will be given to those bearing upon the above-mentioned leading questions. Time will also be allowed sufficient for any demonstrations kindly afforded by the members. The official languages are English, French, German, and Italian. The subscription for membership is one guinea. Subscription forms and further particulars may be obtained from the Hon. Secretary for Great Britain and Ireland, Dr. Arthur Giles, 37, Queen Anne Street, London, W.

